# Factors Influencing the Sponsoring of Animals in Slovak Zoos

**DOI:** 10.3390/ani12010021

**Published:** 2021-12-23

**Authors:** Jana Fančovičová, Pavol Prokop, Róberta Repáková, William Medina-Jerez

**Affiliations:** 1 Department of Biology, Faculty of Education, Trnava University, Priemyselná 4, 918 43 Trnava, Slovakia; jana.fancovicova@truni.sk (J.F.); repakovare@gmail.com (R.R.); 2 Department of Environmental Ecology and Landscape Management, Faculty of Natural Sciences, Comenius University, Ilkovičova 6, 842 15 Bratislava, Slovakia; 3 Institute of Zoology, Slovak Academy of Sciences, Dúbravská cesta 9, 845 06 Bratislava, Slovakia; 4 Teacher Education Department, College of Education, University of Texas at El Paso, El Paso, TX 79968, USA; wjmedinajerez@utep.edu

**Keywords:** donations, ex situ conservation, willingness to pay

## Abstract

**Simple Summary:**

To achieve conservation goals with ex situ programs, zoos have two alternatives: government funding and private donations. By using published data from 2018 on the amount of money received by zoos through adoption programs (if any), we investigated the factors influencing donations in all Slovak zoos. Generalized linear mixed models were applied throughout statistical analyses. Although the majority of animal species in the zoos included in this study had low a conservation status, a few others, like amphibians, were rarely listed as threatened species. In general, vertebrates received more funding than invertebrates, and mammals were the preferred taxa by private contributors. Mammals were sponsored more frequently than non-mammal species, except for reptiles. We submit that zoo managers could concentrate their efforts on the breeding of threatened animals to support their reintroduction to the wild and to enhance people’s awareness of these animal species.

**Abstract:**

Anthropogenic disturbance causes biodiversity loss, and consequently the captive conservation (ex situ) of threatened animals may be an effective strategy in protecting species. We used estimated body mass, phylogenetic closeness with humans, International Union for Conservation of Nature (IUCN) conservation status, and species attractiveness scores, to examine the factors influencing the adoption likelihood of a species in all Slovak zoos. In general, vertebrates received more funding than invertebrates, and mammals were the preferred taxa by private contributors. In terms of funding, we propose that the perception of mammals as phylogenetically close to humans, and attractiveness factor, contribute to an advantage over less attractive and phylogenetically distant species. Conservation status also contributed to the amount of donations; however, the magnitude of these relationships was weak when compared to the effect of animal taxa. These results suggest that Slovak zoos might be more successful in raising donations by breeding threatened species, and raising public awareness about these animal species. Displaying popular, flagship species of non-mammal taxa may increase interest among the public as well, and may translate into a significant growth in the amount of donations.

## 1. Introduction

The human exploitation of Earth’s biodiversity has resulted in continuous habitat loss, environmental pollution and an overall decline in the population sizes of wild species [1]. In response to this issue, efforts to mitigate these losses can be implemented in two ways. First, through in situ conservation, which is defined as the conservation/protection of a species in the wild; second, by pursuing ex situ conservation of a threatened species in captivity. Of the two options, the latter is more commonly implemented in botanical gardens, aquariums and zoos [2].

The main functions of zoos include entertainment, research, education and conservation to achieve the sustainability of populations that rely on our care [3,4]. Nevertheless, reaching these goals constitutes a financial challenge for zoos. Given that zoos and aquariums are visited by about 700 million people annually [5], they serve as suitable places for generating donations to support animal conservation [6]. Although, in general, in situ conservation is more beneficial and less costly [7], ex situ conservation may be very effective in protecting species [8,9,10] to be reintroduced to the wild [11,12]. Indeed, ex situ conservation breeding programs have successfully enhanced the survival of certain species of mammals [13], birds [14], reptiles [15], amphibians [16], and fish [17], which have resulted in high conservation status of these taxa. However, to achieve the sustainability goals of ex situ conservation programs, additional funding and private donations are necessary to support the maintenance of captive species and in situ conversation initiatives [18,19]. In addition, in some cases, in situ programs are not feasible, particularly when habitats have been devastated as a result of armed conflicts [20]. Thus, understanding and taking advantage of zoo visitor preferences for captive animals may significantly increase donation proceeds to support particular species.

The propensity of private individuals to support the conservation of particular species, which could be better utilized in managing ex situ programs, is a scarce line of research in the literature. Moreover, although a good volume of research on sponsorship programs using hypothetical scenarios exist, there is a paucity in studies dealing with actual fundraising programs [21,22,23,24,25,26].

In order to increase the amount of donations, visitor’s passive interest in nature [27] needs to be addressed, particularly by building on the human natural affinity for certain species and/or its status [28,29]. It has been documented that willingness to protect animals is a factor associated with the species’ appearance, and its similarity to humans [30,31,32,33]. For instance, animals that look dangerous [34], disgusting [35], single-colored [25], or unattractive [21,36], receive less conservation support than other animals. Phylogenetic proximity relates to our ability to empathize with animals [37], which seems to trigger our interest in conserving animal species similar to us [30,31,38].

It is also known that human preferences and conservation initiatives are biased toward large-bodied mammals [33,39,40,41,42,43,44], often considered charismatic species. Interest in these species contributes to a bias in the breeding of large-bodied animals in zoos [45,46]. In fact, large-bodied animals are included in zoo collections more frequently than small-bodied animals, irrespective of whether they are threatened or not [12,47,48,49,50,51]. Therefore, one would expect large-bodied, charismatic animals to receive far more donations than smaller, less charismatic species.

The purpose of this study was to determine which animal-related factors influence actual donations that may support fundraising programs in zoos, and ultimately strengthen the effectiveness of conservation marketing [52] of ex situ conservation programs. Willingness levels were measured using actual donations. In this study, we seek to advance this line of research in three different ways: first, our data are not restricted to one zoo, as has been typical in similar research studies. Instead, we used representative, publicly available data on animal sponsorship from all Slovak zoos. Second, we used actual donation figures, rather than hypothetical donations. Third, we did not focus exclusively on sponsored animals, but rather we compared sponsored against non-sponsored animals to better understand which features made the sponsored animals attractive to donors. Fourth, these animal species were compared using a range of key characteristics (phylogenetic distance, estimated body mass, appeal factor and conservation status).

## 2. Materials and Methods

### 2.1. Defining Sponsoring

We analyzed publicly available data published in 2018 in the yearbooks of three Slovak zoos (Bratislava, Bojnice and Spišská Nová Ves) [53,54,55]. The fourth Slovak zoo, in Košice, does not publish yearbooks. In this case, we requested data directly from the zoo headquarters. All yearbooks, including the data from the Košice zoo, contained lists of animal species kept in the zoo, their actual numbers, the conservation status, and information about animal sponsorship. In the context of this study, sponsoring could be defined as the act of willingly transferring personal (institution or company) funds to a conservation organization on an annual basis to sustain a particular species.

Any contribution, large or small, is accepted by the zoos. Funding received from sponsors is predominantly used for species nutrition, and less is used for the reconstruction of the breeding facility, or for the development of the zoo in general. In terms of securing monetary donations, this mostly occurs by people contacting the zoo via e-mail or telephone. When a donation is made, the individual, family, or institution name is added to a placard, which is placed in front of the animal’s enclosure. Each sponsoring period lasts one year, and one species can have multiple sponsors. If an individual donates more than what it is required for a particular species per year, his/her donation is utilized in subsequent years, and the sponsoring is extended. By making the sponsoring status visible to visitors, the zoo underlines a privileged relationship between the sponsoring individuals or institutions and a particular animal species, compared with other non-sponsoring zoo visitors.

Animal adoption programs are common sponsoring strategies; however, other approaches exist. Additional sponsoring methods include donations made by large companies or corporations. In this kind of sponsorship, the contribution does not support a particular species, but the entire zoo. Therefore, hereafter we will use the term “sponsoring” (or “sponsorship”) to refer to animal adoption programs, predominantly made by individuals or small companies. In all these cases, the funds are to be used in the support of specific species. Sponsorship programs are advertised by zoos on their websites. According to the sponsoring individual, family or institution preferences, the zoos allow the sponsoring of different species. Thus, although the actual patterns of sponsorship are partly predetermined by zoos, they also reflect individual preferences. Published yearbooks do not indicate which species were advertised by zoo; thus, we obtained this information directly from the zoos.

**Figure 1:**
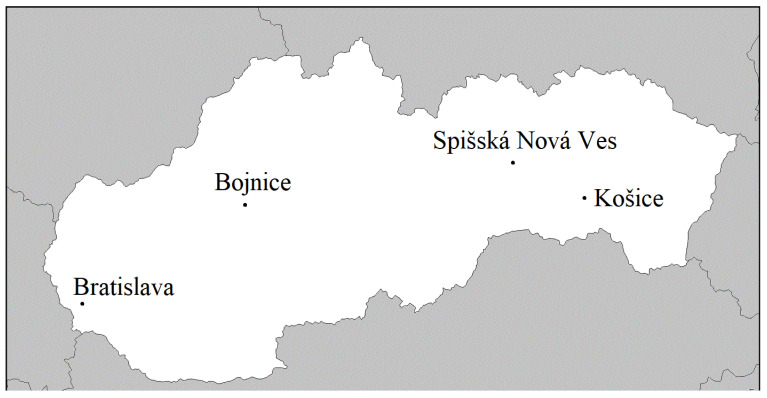
Map of the four Slovak zoos.

The four zoos are located in different Slovak regions (Figure 1). The Bojnice Zoo from the western region is a 42-hectare facility, and is the oldest and most frequently visited zoo (~350,000 people annually); compared with the other zoos, it has the highest species diversity (~400 species, Table 1). The zoo is located in Bojnice, a small town of ~5000 inhabitants, and does not specialize in breeding any specific species. The zoo in Košice is the third largest zoo in Europe (its area is 288 hectares), and is located in East Slovakia in the second largest Slovakian city (~240,000 inhabitants). The zoo is home to about 200 species of animals, and its visitation rate is about 200,000 people per year. The Bratislava Zoo, in the country’s capital city (~450,000 inhabitants), has an area of 96 hectares. It has more than 150 animal species. It also has a high visitation rate, approximately 300,000 people annually. The zoo in Spišská Nová Ves is the smallest of the four zoos (8 hectares) included in this study, and is located in East Slovakia, with about 100 species of animals. The city has about 40,000 inhabitants. It has the lowest visitation rate: about 100,000 people per year come to visit this zoo.

### 2.2. Measured Variables

#### 2.2.1. Sponsoring (Dependent Variable)

Sponsoring was examined using the following variables: total and mean contribution amounts for all species per zoo in 2018 (in Euros) and sponsorship frequency in 2018. Whether the sponsoring occurred or not was defined as a binary variable.

#### 2.2.2. Conservation Status (Independent Variable)

The conservation status of all listed animals in each zoo was derived from IUCN categories [56]. For statistical purposes, we transformed these categories into a threatened/non-threatened binary variable, where threatened corresponds to VU, EN and CR as per IUCN terminology.

#### 2.2.3. Body Mass and Phylogenetic Closeness with Humans (Independent Variable)

The body mass of each species was calculated using data from Jones et al. [57]. Data were log-transformed, following the recommendations from Smith et al. [42]. The phylogenetic divergence time from humans (in millions of years) was obtained for each species from timetree.org [58]. A few specimens were apparently hybrids, or their scientific names were incorrect. In such cases, body mass or phylogenetic closeness data were omitted.

#### 2.2.4. Appealing Species (Independent Variable)

The appealing species factor was defined according to the appeal scores available for 4320 species of mammals [59]. These scores express participants’ preference for each species in the context of conservation. Higher scores mean greater appeal (range = −0.77–5.01). Further details about the score can be found in MacDonald et al.’s (2017) study [59].

### 2.3. Statistical Analysis

The data were analyzed using Generalized Linear Mixed Models (GLMMs) where zoo and species identity were defined as random factors. Dependent variables were binomial (occurrence of sponsoring or not) or continuous (amount of donated money) with Poisson distribution. Data were checked for normality using the Kolmogorov–Smirnov test. As at least some of them were not normally distributed, additional comparisons of body mass and species appeal scores between threatened and non-threatened species were performed with the Mann–Whitney U-test. Correlations between species appeal score, body mass, phylogenetic closeness and financial support were performed with the Spearman correlation coefficient. It is important to note that in this study, sample sizes varied due to the lack of data on conservation status, body mass or appeal scores for certain species. We mainly investigated sponsored species in comparison to all non-sponsored species residing in zoos. In order to address whether possible differences could be confounded by species advertised on zoo web pages, we ran additional analyses with a subsample of advertised species. Statistical tests were performed in SPSS version 23.

## 3. Results

### 3.1. Sponsoring of Vertebrates versus Invertebrates

The total number of species across all zoos was 813. Of this number, nearly 25% of the species (N = 237) were reported in more than one zoo. A combined total of 1055 animal species were listed in all four zoos (Table 1, Figure 1).

Considering reports from all the four zoos, vertebrates (148/984, 15%) were sponsored more frequently than invertebrates (1/71, 1.4%) (GLM with binomial distribution of data, Walds χ^2^ = 8.1, df = 1, *p* = 0.004). Of the 813 animal species in the four zoos, the Emperor scorpion (*Pandinus imperator*) was the only sponsored invertebrate species. Vertebrates were sponsored exclusively by individuals (57%), corporations (33%) or both (10%). As there were no significant differences between vertebrate classes being sponsored by individuals, corporations or both groups (Chi-square test, χ^2^ = 3.8, df = 6, *p* = 0.7), we are not referring to sources of sponsorship in subsequent analyses.

Considering reports on all 682 animals advertised on zoo web pages, once again, vertebrates (148/662, 22%) were sponsored more frequently than invertebrates (1/26, 4%). This difference certainly approached significant levels (*F*_2,571_ = 2.85, *p* = 0.059).

Sponsoring occurrences were more frequent in Bratislava (50%), followed by Spišská Nová Ves (24%), in Košice (18%) and Bojnice (5%). Therefore, we defined the effect of zoo location (city) along with species ID as random factors in the subsequent statistical analyses. Taking into account that the sponsoring rate of invertebrates was exceptionally rare, we made the decision to continue with analyses exclusively on vertebrates.

#### 3.1.1. Vertebrate Sponsorship

**Figure 2:**
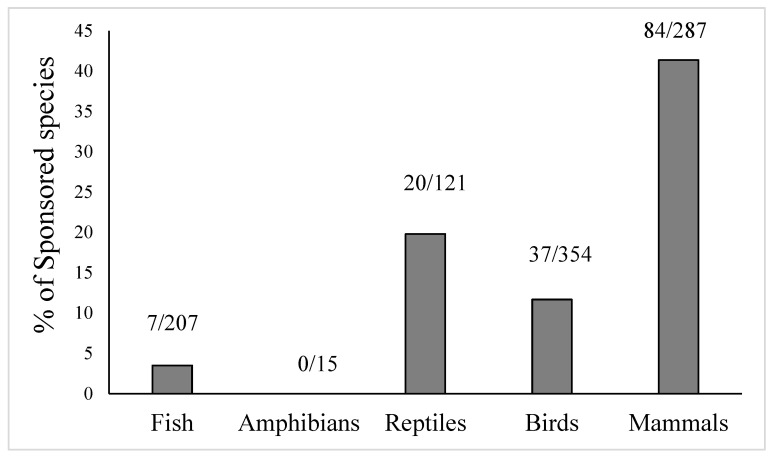
Distribution of sponsoring occurrences among vertebrate classes.

Vertebrate classes and conservation status were predictors in the GLMM model; species ID and zoo were random factors, and the occurrence of sponsoring was the dependent variable. The model was significant (*F*_5,769_ = 3.56, *p* = 0.003). Vertebrate classes (*F*_4,769_ = 4.22, *p* = 0.02), but not conservation status (*F*_1,769_ = 3.0, *p* = 0.08), played a significant role in sponsoring occurrence. The interaction term was not statistically significant (*F*_4,765_ = 0.26, *p* = 0.90). The effect of the vertebrate class factor was very clear. Mammals were sponsored more frequently than non-mammal species (all *p* < 0.01), except for reptiles (*p* = 0.07) (Figure 2). When the total sponsoring number (range: 0–7, mean = 0.24, SE = 0.02, N = 984) was set as a dependent variable, the model was significant (*F*_9,765_ = 2.74, *p* = 0.004), but neither vertebrate class (*F*_4,765_ = 2.02, *p* = 0.09), nor the conservation status (*F*_1,765_ = 0.38, *p* = 0.54), or the interaction term (*F*_4,765_ = 0.46, *p* = 0.76) showed any significant influence on the dependent variable.

When we considered only a subset of species that were advertised on zoo websites, the results were almost identical; the model was significant (*F*_6,549_ = 2.24, *p* = 0.038). Vertebrate classes (*F*_5,549_ = 2.65, *p* = 0.02), but not conservation status (*F*_1,549_ = 0.03, *p* = 0.87), played a significant role in sponsoring occurrence. The interaction term was not statistically significant (*F*_4,545_ = 0.25, *p* = 0.91).

#### 3.1.2. Amount of Financial Support by Animal Class

By looking at the large amounts of money contributed to the mammal class, our previous analysis suggests that in terms of sponsoring occurrences, this was the preferred class among vertebrate animals. In subsequent analyses, we calculated whether there were any differences in the mean amount of financial support among vertebrate classes in both sponsored and non-sponsored animals. The GLMM with Poisson distribution was set with the same predictors as in the previous analysis, and with the mean amount of money contributed per species as the dependent variable. The model was significant (*F*_9,644_ = 698.4, *p* < 0.001). The vertebrate class was a strong predictor of the mean amount of proceeds (*F*_4,644_ = 1367.4, *p* < 0.001). Contrast analysis between vertebrate classes showed that mammals received the greatest amount of donations compared to all the other vertebrate classes (all *p* < 0.001, Table S1). Species with a high conservation status received greater support than those with a lower conservation status (*F*_1,644_ = 94.1, *p* < 0.001). The interaction term was significant (*F*_4,644_ = 148.7, *p* < 0.001), suggesting that threatened mammals received greater support than non-threatened mammals. In contrast, no differences between animals with low and high conservation status among remaining vertebrate taxa were observed.

When considering advertised species, the results were almost identical (whole GLMM model, *F*_7,96_ = 771.8, vertebrate class, *F*_4,96_ = 768.9, conservation status, *F*_1,96_ = 1135.5, interaction term, *F*_2,96_ = 232.4, all *p* < 0.001).

When the *total* amount of money was defined as the dependent variable, the model remained significant (*F*_8,572_ = 934.6, *p* < 0.001). Again, vertebrate class (*F*_4,572_ = 1835.6, *p* < 0.001) and conservation status (*F*_1,572_ = 444.4, *p* < 0.001), along with the interaction term (*F*_3,572_ = 21.3, *p* < 0.001) were significant predictors of total donation amount. Again, mammals received more donations than other vertebrate classes (analysis of contrasts, all *p* < 0.001). The top 10 mammal and bird species with the highest mean amount of donated money are shown in Table 2.

Considering the advertised species, results were once again almost identical (whole GLMM model, *F*_7,103_ = 1545.8, vertebrate class, *F*_4,103_ = 1865.4, conservation status, *F*_1,103_ = 579.9, interaction term, *F*_2,103_ = 70.8, all *p* < 0.001).

#### 3.1.3. Factors Influencing the Sponsoring of Mammals

We conducted an analysis restricted to mammals, with occurrence of sponsoring as the dependent variable, and conservation status, body mass, phylogenetic closeness to humans and species appeal scores as predictors, and the ID of species and zoos as the random factors. The GLMM model was not significant (Table 3). Although all main effects were non-significant, the interaction term Conservation Status × Species Appeal certainly approached significance (Table 3). This finding suggests that the appeal scores of sponsored threatened species were higher than those of sponsored, but non-threatened, species. In contrast, there was a small difference in appeal scores in non-sponsored threatened or non-threatened mammals.

The GLMM with a subset of advertised species was not statistically significant (*F*_8,142_ = 0.99, *p* = 0.44, all *p* > 0.14 for fixed effects including the interaction terms).

#### 3.1.4. Additional Relationships between Sponsoring and Species Characteristics

It was also noted that body mass had a positive and moderate correlation with the appeal score (Spearman *r* = 0.42, *p* < 0.001, N = 177) and that threatened species showed higher appeal scores (median = 2.23, 95% CI [2.02, 2.55], N = 76) than non-threatened species (median = 0.6, 95% CI [0.43, 0.71], N = 161) (Mann–Whitney U-test, U = 9039, *p* < 0.001). Although threatened species tended to be heavier (median = 154.3 kg, 95% CI [14, 324.1], N = 58) than non-threatened species (median = 10 kg, 95% CI [32.6, 68.3], N = 125), this difference was not significant (Mann–Whitney U-test, U = 4045.5, *p* = 0.23).

Correlations and comparison with a subset of advertised species showed almost identical results as in the cases above mentioned.

Spearman correlations did not show significant associations between the mean or total amount of financial support and species body mass, phylogenetic closeness, or appeal score (all *p* > 0.2). The same results were obtained with a subsample of the advertised species.

## 4. Discussion

### 4.1. Phylogenetic Closeness to Humans

We investigated the relationship between donations and species phylogenetic closeness to humans at various levels. First invertebrates, which are phylogenetically distant from humans, were sponsored at lower rates compared with those species phylogenetically closer to vertebrates. This result corroborates what prior research studies have found, that people have negative attitudes and perceptions towards invertebrates [33,60,61,62,63,64]. Low conservation status of invertebrates in Slovak zoos, and to certain extent, their lack of promotion campaigns among the general public contribute to being ignored in sponsoring programs. It is possible that zoos do not engage in fundraising in favor of invertebrate species through sponsorship programs. We submit that flagship species, such as butterflies, dragonflies or corals, which are highly regarded by zoo visitors [44,63,65], should be used to increase the awareness of these species among the public and the willingness to support their conservation.

Second, across animal orders, mammals that are phylogenetically closer to humans had higher sponsoring rates than phylogenetically distant vertebrates. Thus, the likeability or appeal of mammals at the expense of other taxa plays a role in species preferences [66]. A global analysis focused on crowdfunding platforms also showed that mammals, followed by birds, received more funding than other animal taxa [67]. When the relationship between phylogenetic distance and the likelihood of sponsoring/amount of donations were analyzed (exclusively within the mammal class), the correlation was not significant as was expected, according to the phylogenetic closeness hypothesis. In particular, we noted that the likelihood of a species being sponsored was weakly associated with phylogenetic closeness to humans. Although the amount of monetary contributions was greatly influenced by phylogenetic closeness [26], the magnitude of this relationship was much weaker when compared with species appeal. These results suggest that the willingness of an individual or organization to commit to an annual sponsoring contribution in Slovak zoos can be only partly explained by phylogenetic closeness. However, certain animal physical attributes seem to be relevant when securing annual sponsorships. It should be noted that “similarity” does not necessarily mean the same as “phylogenetic closeness”. For instance, a chimpanzee, the phylogenetically closest mammal to humans is much less preferred by children than parrots or dolphins [64], despite both parrots and dolphins being more phylogenetically distant to humans than chimpanzees. In fact, neither chimpanzees nor orangutans, as the phylogenetically closest species to us (note that gorillas, another phylogenetically close relative, are not breed in Slovak zoos) were sponsored in Slovak zoos. Perhaps this can be explained on the basis of the perceived beauty of the parrots [48] and the playful and altruistic nature of parrots and dolphins (unlike chimpanzees) that make them popular and attractive species in zoo collections [68]. This factor should be taken into consideration in future assessments of human–animal relationships, to better understand the willingness of individuals and organizations to become involved in species conservation programs.

### 4.2. The Effect of Species Appeal and Body Size

Appealing species were no more frequently sponsored, and did not receive greater amounts of donations than less-appealing species. This finding was also reported in a recent study conducted in Finland [69]. Moreover, body mass of mammals had a positive correlation with their appeal score, and threatened species showed higher appeal scores than non-threatened species. This finding is in correspondence with the overall human affinity towards large charismatic animals [39,40,70]; however, the willingness to support conservation efforts focused on these species [23,26,44,71] was not confirmed. Some researchers suggested [72,73] that these charismatic species would be beneficial by maximizing shared benefits with other species. This feature can also reflect the neglect of non-charismatic/Cinderella species in zoos [29,42,74].

### 4.3. The Effect of Conservation Status

Species conservation status played a significant, but not prominent role in sponsoring, and in the amount of donations. Our analyses suggest that endangerment of vertebrates did not automatically result in sponsoring occurrences. Moreover, threatened mammals were not likely to be sponsored, compared with non-threatened mammals. Regarding mean donation amounts, the conservation status of vertebrates was a significant factor, but this was much weaker than vertebrate class, with mammals receiving most of the donations. Similar results were reported in another study [59] in which appealing species did not necessarily have a high conservation status. Sponsored threatened mammals tended to be more attractive when compared with sponsored non-threatened or non-sponsored (threatened or not) mammals, which once again suggests that species attractiveness and conservation status play a non-trivial role in sponsoring decisions. In terms of sponsoring occurrence, these findings do not lend significant support to the assumption that people seem to prefer large-bodied over small-bodied vertebrates [39,40,41,70].

The majority of animals in the four Slovak zoos had low conservation status (20% of vertebrates with an IUCN status were threatened). This was true for invertebrates, fish and, in particular, amphibians. These taxa are globally neglected, not only by zoo visitors [66], but also in fundraising programs taking place around the globe [67]. It is apparent that zoos did not value the conservation priority of amphibians, which are subject to captive breeding [75]. This trend reflects the low numbers of amphibian species in European zoos [76]. We propose that increasing the proportion of endangered species and the use of aesthetically appealing amphibians in advertisement [77] can contribute to an increase in sponsorship rates, and ultimately strengthen the investment capacity of zoos. A similar approach can be applied to other neglected taxa.

In a survey with Australian participants, the authors found that people preferred in situ management of wild populations of native bird species, over captive breeding [78]. In fact, it is recommended that zoos consider an in situ–ex situ combined approach in their conservation activities [12]. Employing in situ conservation can be complicated, as determined by the strong preferences for exotic animals over native ones [79], except for vertebrates including the brown bear, the wild boar and the golden eagle. The remaining 17 species listed with top mean contributions (Table 1) were exotic. Out of these 20 species, only the golden eagle has an endangered status, and we suggest it could be used for in situ programs directly in Slovakia. Therefore, zoos can benefit from designing and implementing management strategies that promote public interest in threatened native species, and develop purposeful campaigns to generate monetary donations.

### 4.4. Gaps and Recommendations for Future Research

Animal collections in zoos are predominantly focused on popular or well-known and non-threatened animals. We propose that zoo managers could concentrate their efforts on the breeding of threatened animals to support their reintroduction to the wild and to enhance people’s awareness of these animal species. Future research could involve sponsoring individuals and organizations to gather information regarding their decision to participate in sponsoring programs.

## 5. Conclusions

In conclusion, increasing the number and amounts of donations in Slovak zoos through sponsoring programs is an initiative largely influenced by animal taxa. Moreover, motives are partially influenced by animal conservation status and by species attractiveness, rather than by animal size. In all probability, individuals and organizations’ willingness to sponsor animals in zoos is multidimensional [80]. Displaying popular, flagship species of non-mammal taxa may also raise public awareness and interest, and in turn increase the amount of donations. Interviewing individuals and organizations participating in sponsoring campaigns (and those who did not) may provide further insights into individuals’ motivation to sponsor animals in zoos.

## Figures and Tables

**Table 1:** Reported number of species in the four Slovak zoos.

	Class	Bratislava	Bojnice	Spišská N. Ves	Košice
Invertebrates	Anthozoa	0	7	6	9
	Arachnida	2	2	3	2
	Insecta	6	0	1	12
	Mollusca	3	0	0	6
	Asteroidea	0	0	0	2
	Echinoidea	0	0	0	1
	Crustacea	0	0	0	3
	Polychaeta	0	0	0	5
	Polyplacophora	0	0	0	1
Vertebrates	Fish	20	110	25	52
	Amphibia	0	15	0	0
	Reptiles	20	50	13	38
	Birds	47	165	34	108
	Mammals	83	81	47	76

**Table 2:** Mammals and birds with top mean contributions.

	Amount of Money (€)			Amount of Money (€)	
Mammals	Mean	Total	Birds	Mean	Total
Jaguar (*Panthera onca*)	3000	3000	Black swan (*Cygnus atratus*)	4550	8100
Lion (*Panthera leo*)	1640	1640	Humboldt penguin (*Spheniscus humboldti*)	400	800
Tiger (*Panthera tigris*)	1330	1330	Great flamingo (*Phoenicopterus roseus*)	350	700
Brown bear (*Ursus arctos*)	1262.5	5050	Golden eagle (*Aquila chrysaetos*)	235	470
Pony (*Equus ferus*) *	1200	1200	Scarlet ibis (*Eudocimus ruber*)	208	416
Black lemur (*Eulemur macaco*)	1001	1001	Green-billed toucan (*Ramphastos dicolorus*)	200	200
Wolf *(Canis lupus)*	725	1450	Canada goose (*Branta canadensis*)	185	185
Donkey (*Equus asinus*)	666	666	Snowy owl (*Bubo scandiacus*)	155	785
Cougar (*Puma concolor*)	665	665	Emu (*Dromaius novaehollandiae*)	115	180
Wild boar (*Sus scrofa*)	500	500	Red-winged parrot (*Aprosmictus erythropterus*)	103.3	310

* Domestic animals were not included in the statistical analyses because they lack IUCN conservation status.

**Table 3:** Results of GLMM in relation to sponsoring occurrence in mammals.

	F	df1	df2	*p*
Corrected model	0.869	10	152	0.564
Conservation status (CS)	0.389	1	152	0.534
Body mass	1.487	1	152	0.225
Phylogeny	0.279	1	152	0.598
Appeal	0.597	1	152	0.441
CS × Body mass	1.57	1	152	0.212
CS × Phylogeny	0.683	1	152	0.41
CS × Appeal	3.798	1	152	0.053
Body mass × Phylogeny	0.851	1	152	0.358
Appeal × Body mass	0.042	1	152	0.838
Appeal × Phylogeny	0	1	152	0.989

## Data Availability

Data are available in corresponding author upon reasonable request.

## Supplementary Materials

The following are available online at https://www.mdpi.com/article/10.3390/ani12010021/s1, Table S1: List of sponsored animal species.
